# Is Herpes Simplex virus (HSV) a sign of Encephalitis in Iranian Newborns? Prevalence of HSV Infection in Pregnant Women in Iran: A Systematic Review and Meta-Analysis

**Published:** 2017

**Authors:** Masoumeh ARABSALMANI, Meysam BEHZADIFAR, Hamid Reza BARADARANMD, Mansoureh TOGHAE, Gholam BEYRANVAND, Alireza OLYAEEMANESH, Masoud BEHZADIFAR

**Affiliations:** 1 Department of Health, Faculty of Health, Zahedan University of Medical Sciences, Zahedan, Iran.; 2 Social Determinants of Health Research Center, Lorestan University of Medical Sciences, Khorramabad, Iran.; 3 Department of Epidemiology, Faculty of Health & Nutrition, Lorestan University of Medical Sciences, Khorramabad, Iran.; 4 Endocrine Research Center Institute of Endocrinology and Metabolism, Iran University of Medical Sciences, Tehran, Iran.; 5 Department of Neurology, Sina Hospital, Tehran University of Medical Sciences, Tehran, Iran.; 6 Department of Health, Faculty of Health, Lorestan University of Medical Sciences, Khorramabad, Iran.; 7 National Institute for Health Research,Tehran University of Medical Sciences,Tehran,Iran.; 8 Health Management and Economics Research Center, Iran University of Medical Sciences, Tehran, Iran.

**Keywords:** Herpes simplex virus, Pregnancy, Meta-analysis, Iran

## Abstract

**Objective:**

Herpes Simplex virus (HSV) is one of the most common sexually transmitted diseases in the world. This study aimed to determine the prevalence of herpes simplex virus in pregnant women in Iran.

**Materials & Methods:**

A systematic literature review was conducted to study the HSV subtypes in Persian and English papers through several databases. We searched Pub Med, Scopus, Ovid, Science Direct and national databases as Magiran, Iranmedex and Science Information Database (SID) up to October 2015. Random-effects model were applied to calculate the pooled prevalence of HSV subtypes.

**Results:**

Five eligible studies were identified, including 1140 participants. The pooled prevalence of HSV infection in pregnant women was 0.64% (95% CI: 0.10- 1.18) in Iran. The pooled prevalence of studies on both HSV-1 and HSV-2 was 0.91% (CI: 0.81-1.02) and studies on only HSV-2 was 0.23% (CI: -0.61-0.63), respectively.

**Conclusion:**

The prevalence of HSV infection in pregnant women in Iran was higher. HSV infection of the central nervous system, especially with HSV-2, can also cause recurrent aseptic meningitis and monophasic, as well as radiuculitis or myelitis. The performance of screening to detect infection in pregnant women can play an important role in the prevention and treatment of patients and help to prevent the transmission of HSV infection to infants in Iran.

## Introduction

Herpes simplex virus (HSV) infections are very common worldwide(1).They are caused by either HSV-1 or HSV-2, and the majority is asymptomatic(2). HSV-2 prevalence among pregnant women has been estimated as 20%-30%, with approximately 10% of HSV-2 seronegative women living with a seropositive partner and hence, at risk in the acquisition of herpes during pregnancy (3, 4). The potential factors associated with HSV-2 infection are age and sex or gender. Ageing increases the risk of HSV (5). Other factors, such as having sexual intercourse sooner than common age and risky sexual relationship, poverty, gender or ethnicity, and bacterial vicinities can facilitate women’s risk of infection before their pregnancy (6, 7).In the US, 22% of pregnant women are infected with HSV-2.

Among whom two percen tare infected during pregnancy,which threatens them and their baby (8).Some studies have demonstrated the prevalence of HSV type 1 or 2 during pregnancy(9, 10).Nearly 30%-50% of babies can be infected with the virus and at the end of pregnancy period; the risk of infection may increase (11).

The problem is not just relatively high prevalence of HSV infection among pregnant women, but unawareness of this infection, which affects newborns drastically (1, 12). About three-fourth of women with a history of HSV show incidence of HSV again during their pregnancy and one out of seven suffer from lesion through delivery(13,14).

HSV infections during pregnancy may cause fetal and neonatal infections. HSV transmission may occur during pregnancy and after delivery, 80%-90% of neonatal herpes infections occur when the baby passes through the mothers’ infected birth canal. HSV infection can have severe consequences for the affected newborns. HSV infection may turn to herpes encephalitis or infection across other organs such as liver, lungs, and kidneys with or without dermal symptoms. 

HSV causes disordering of some parts of the body, such as CNS, skin, eyes, lungs, mouth, adrenal gland and liver, and in the absence of therapy, it has a mortality risk of about 80% (15).Babies with the disease usually die because of viral pneumonia and intravascular coagulopathy. Survivors (babies who survived) from serious infection generally suffer from permanent neurological disorder (16-19). This study aimed to determine the prevalence of HSV infection in pregnant women in Iran.

## Materials and Methods

**Study eligibility and identification**

We investigated to estimate the rate of HSV infection rates among Iranian pregnant women in Iran and the neurologic impact of HSV on the newborns. A literature search was performed in which PubMed, Scopus, Ovid and Science Direct as well as national databases such as MagIran, Iranmedex and Science Information Database (SID) using “Prevalence” OR “frequency” AND “herpes simplex virus” OR “HSV”OR “TORCH”AND “pregnancy” AND “Iran “as keyboards up to October 2015.In addition, the reference lists of several studies and conferences related to the present study were investigated.

**Inclusion and exclusion criteria**

Studies that report on the prevalence of HSV among pregnant women in Iran were included while Case Reports, Case Series and Quasi – experiments and studies whose methods and results were not clear and the population did not comprise of Iranian pregnant women were excluded.

**Data extraction**

Independent literature searches were conducted by two reviewers and experts and after omitting the repeated cases. They chose the title and abstract studies based on the inclusion and exclusion criteria. The name of authors was blinded. If there was a problem or disagreement between two investigators, the third one helped them to solve the problem by discussion. The name of first author, the year of publication of article, the amount of sample, the number of positive virus, the type of virus and the place of studies extracted were analyzed.

**Quality assessment of studies**

To assess the quality of the studies, the STROB checklist was used(20). The studies were classified as high, intermediate and low quality.

**Statistical analysis**

The data obtained from the various studies were analyzed using a random effect with 95% confidence intervals by inverse variance weight. The heterogeneity between the included studies was assessed using Q-test and I2 index.

By so doing, a P-value of less than 0.01% was considered as significant in the heterogeneity test. Thereafter, subgroup analysis was carried out based on virus type, year of publication and quality of studies. All analyses were performed using Review Manager Version 5.3 software. Assessment of publication bias was planned by visualizing the funnel plot asymmetry. However, since the number of included studies was less than the recommended optimum number of at least 10, the assessment of publication bias was not carried out (21).

## Results

**Fig 1:**
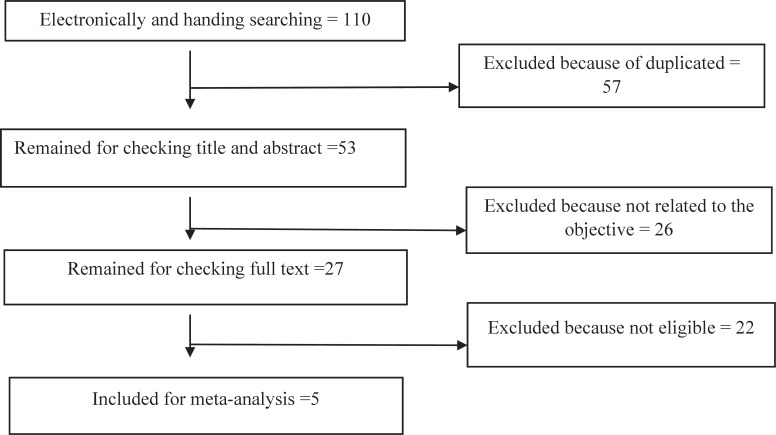
Flow chart selected studies for meta-analysis

Overall, 110 articles were obtained and after omitting the repeated studies, 53 articles were left. Based on analysis, the titles and summaries of 27 articles were selected and their texts were completely analyzed. In addition, after the evaluation of all articles, five of the articles were selected (22-26).Some studies were omitted for reasons such as lack of suitable and useful factors like poor design or lack of suitable design, and poor results or appropriate population. Figure 1 shows the process involved in the search and finding of the studies. Five studies were included for meta-analysis. In addition, studies carried out during 2003 to 2015 involving 1140 participants were included. Table 1 shows the characteristics of the studies.

**Fig 2:**
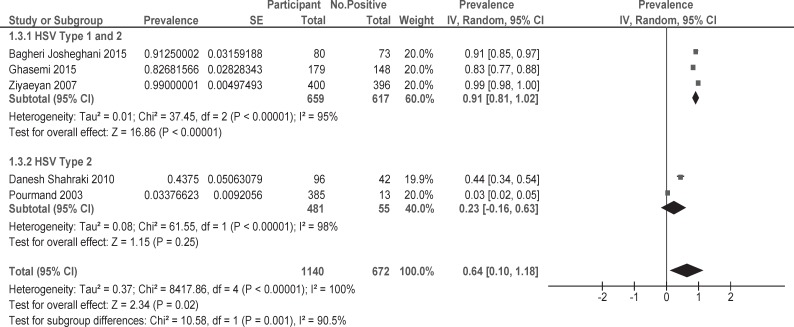
Overall prevalence of HSV infection in pregnant women in Iran

The overall prevalence of HSV infection in pregnant women in Iran was0.64% (95% CI: 0.10-1.18) (Figure 2). The high prevalence was observed in Tehran Province with 0.99 % (95% CI: 0.98- 1.00) and low prevalence was observed in Kermanshah Province 0.03% (95% CI: 0.02-0.05), respectively. Moreover, based on different types of HSV, studies which involved both types 1and 2 prevalence was 0.91% (95% CI: 0.81, 1.02) and studies that involved only type 2 prevalence was 0.23% (95%

CI: -0.16-0.63) respectively.

**Quality assessment of the studies**

Based on analyzing the quality of studies, which included meta-analysis according to the STROB checklist,two studies were of high quality, two studies were of intermediate quality, and a study was of low quality. 

HSV prevalence was calculated based on the quality of studies. Prevalence in studies with higher quality was more than the overall prevalence. In addition, prevalence in studies with lower quality was less than overall prevalence.

**Table 1:** Characteristics of the Studies in Meta-Analysis

**Author**	**Year**	**Location**	**Sample size**	**Type HSV**
**Ziayian et al** **.**	2007	Tehran	400	Type 1and 2
**Pourmand et al** **.**	2003	Kermanshah	385	Type2
**Danesh shahraki et al.**	2010	Esfahan	96	Type2
**Bagheri joshaghani et al.**	2015	Kashan	80	Type 1and2
**Ghasemi et al.**	2015	Tehran	179	Type 1and2

**Table 2:** Subgroup Analysis HSV Infection in Pregnant Women in Iran

	No studies	Prevalence	CI 95 %	*P* value
**Year of publication**				
Before 2008	3	0.51	-0.43 to 1.45	0.284
After 2008	2	0.73	0.50 to 0.96	0.000
**Quality of the studies**				
High	2	0.87	0.78 to 0.95	0.000
Intermediate	2	0.72	0.17 to 1.26	0.010
Low	1	0.03	0.02 to 0.05	0.000
**Location of the studies **				
Tehran	2	0.91	0.75 to 1.07	0.000
Other cities	3	0.46	-0.16 to 1.08	0.143
**Sample size **				
< 200	3	0.73	0.50 to 0.96	0.000
> 200	2	0.51	-0.43 to 1.45	0.284

**Subgroup analysis**

Table 2 shows the result of subgroup analysis based on the quality of studies, year of publication, place of the studies and sample size.

## Discussion

HSV infection is one of the public health problems around the world, which infect people yearly. This virus poses significant problems for infected people. During pregnancy, this virus can be dangerous to the fetus and cause irreparable damage (11).This study is by our best of knowledge the first systematic review and metaanalysis on the prevalence of HSV infection among Iran’s pregnant women.

The prevalence of HSV was observed as 0.64%. HSV prevalence based on studies of both HSV-1 and HSV-2 was observed as0.91% and studies on only HSV-2 was observed as 0.23 %(Fig.2). Nevertheless, the primary statistics show that prevalence of HSV in Iran is higher than that developed nations and lower in comparison with other developing ones (27, 28).

Central nervous system disease alone occurs in one-third of all infants with neonatal herpes simplex virus infection (29). Localized herpes simplex virus have been found in the fifthly percent of the affected neonates, involvement of the central nervous system in the thirty three percent, and disseminated infections in the seventeen percent (30-31). Although HSV-1 has a predilection for the development of encephalitis after intracerebral injection in the mouse model, HSV-2 generally causes meningitis. 

However, the meninges are not the only component of the central nervous system involved in HSV-2 infection (32). Virtually any part of the neuraxis may be affected by this virus, including the brain, brain stem, spinal cord, nerve roots, cranial nerves and retina. HSV infection of the CNS, especially with HSV-2, can also cause recurrent aseptic meningitis and monophasic, as well as radiuculitis or myelitis (33). When HSV-2 infection is mentioned, neonatal herpes simplex encephalitis, a devastating disorder, is the disease most commonly considered. Seventy percent of affected neonates are born to mothers without symptoms or signs of genital herpes (34). Recent studies suggest that as much as 30% of neonatal herpes simplex encephalitis is due to HSV-1. The risk of acquisition during a primary infection with HSV-1 or HSV-2 is the fifthly percent (34). The risk of development of neonatal HSE is reduced if a mother with primary HSV-2 genital herpetic infection is seropositive for HSV-1. Risk factors for neonatal HSV disease include first-episode maternal infection in the third trimester, invasive monitoring, and delivery before a gestational age of 38 weeks, and maternal age of less than 21 years. Delivery by cesarean section significantly reduces the risk of HSV acquisition (35).

Tehran has significantly the most prevalence of HSV in Iran. The high prevalence in Tehran can be attributed to the high population density of Tehran, numerous poor areas around its territory, different cultural conditions, different thnicities, precocious puberty in children, high risk behaviors, immigration and poverty of the city.

On the other hand, unhealthy lifestyle, lack of following primary health care, turning from helpful traditions and believes as well as low quality of life in other areas can result in high prevalence of HSV(36-37).

Iran is a developing country with a young population and many people on the verge of marriage. Some reasons, such as sparse population in vast area, bordering with Afghanistan and Iraq and many migrations from these countries to Iran, have changed sexual activities in the country. Diverse race types and cultures in Iran could also possibly foster the high prevalence of HSV (38). Although, these conditions are risky factors for high infection by HSV, no study has investigated the relationship between these factors and more infection by HSV.

A high heterogeneity was observed between the accepted studies and those analyzed based on the amount of P-value, statistical tests I2and Chi – Square.

This heterogeneity can be attributed to the number and sample size (quantity of the sample). The metaanalysis of studies is another reason for this statistically significant difference. In addition, the prevalence of this virus in high-qualified studies and low prevalence in low qualified studies could be another reason(39). 

In this present study, some limitations were observed.Firstly, the quality of the articles, and the fact that 20% of the articles were of low quality could affect the results of this study. Secondly, the low number of systematic reviewers of studies and, thirdly the high number of articles studied in Tehran may cause bias in the results of this study.

**In conclusion, **the overall prevalence of HSV infection in pregnant women in Iran was observed as 0.64%. In recent years, the prevalence of HSV in pregnant women has increased. However, the high prevalence of HSV has also been demonstrated among young Iranian population.

Improvement in the standard of living, which increases health activities, should be facilitated by the primary health care (PHC) providers and this can be effective in improving the health status of the individual. In addition, screening to detect infection in pregnant women can play an important role in the prevention and treatment of patients and in the prevention of its transmission to infants in Iran. The high HSV infection in pregnant women is a significant challenge for disease control and surveillance in Iran.
